# Complex skin cancer treatment requiring reconstructive plastic surgery: an interview study on the experiences and needs of patients

**DOI:** 10.1007/s00403-021-02204-3

**Published:** 2021-02-21

**Authors:** Sven van Egmond, Marlies Wakkee, Marit Hoogenraad, Ida J. Korfage, Marc A. M. Mureau, Marjolein Lugtenberg

**Affiliations:** 1 Department of Dermatology, Erasmus MC Cancer Institute, Rotterdam, The Netherlands; 2 Department of Public Health, Erasmus MC, Rotterdam, The Netherlands; 3 Department of Plastic and Reconstructive Surgery, Erasmus MC Cancer Institute, Rotterdam, The Netherlands

**Keywords:** Skin cancer, Reconstruction, Patient-centered, Needs, Experiences

## Abstract

To provide patient-centered care, it is essential to explore what patients consider important and to adjust care accordingly. This may specifically be relevant for patients with complex skin cancer, for whom the care process is often more complicated and psychological and social problems may play a larger role. The objective was to explore the experiences and needs of patients who had undergone surgical treatment by a dermatologist for a complex skin cancer with a subsequent reconstruction by a plastic surgeon. An interview study was conducted among 16 patients who had undergone surgical treatment by a dermatologist and reconstruction by a plastic surgeon for basal cell carcinoma, cutaneous squamous cell carcinoma, or lentigo maligna. The interviews focused on patients’ experiences and needs regarding care using a predefined topic list. All interviews were audio-taped, transcribed verbatim and inductively analyzed using Atlas.ti. Patients reported a need for a skilled and friendly physician who tailors information and communication to their individual situation. A need for continuity of care and improved collaboration between healthcare providers was also emphasized. Furthermore, patients experienced complications and unmet expectations and expressed a need for shared decision-making at various steps throughout the treatment process (depending on age). Patients also considered completeness of tumor removal, follow-up visits with multiple specialists to be planned the same day and recognition of the psychological impact of the disease on the partner important. To improve patient-centered care for complex skin cancer patients, more efforts should be directed towards improving continuity of care and collaboration. Furthermore, it is advocated for physicians to be sensitive to the individual needs of patients and their partner and adjust information, communication and (supportive) care accordingly.

## Key points for decision makers

- The healthcare regarding patients with a complex skin cancer is complicated as they are treated multidisciplinary team and susceptible to social and psychologic problems.
- In this qualitative study, 16 skin cancer patients were interviewed who underwent surgery by a dermatologist and subsequently reconstruction by a plastic surgeon regarding their experiences and needs.
- More efforts should be undertaken to improve continuity of care and collaboration between healthcare providers to improve patient-centered care for complex skin cancer patients. To meet patients’ needs, physicians should adapt their information, communication and care to the individual patient and their partner.

## Introduction

Basal cell carcinoma (BCC), cutaneous squamous cell carcinoma (cSCC) and lentigo maligna (LM) are among the most frequent (pre)malignancies of the skin, with increasing incidence worldwide [1–3]. BCC and cSCC are subtypes of non-melanoma skin cancer and LM is considered a precursor of LM melanoma [3, 4]. The main treatment modality of loco(regional) skin cancer is surgery, which in most cases is performed by dermatologists [5–8].

In patients with complex skin cancer, the lesion is usually located at the scalp or face, making it challenging to remove and reconstruct due to size, location and/or depth [9, 10]. This group predominantly not only consists of elderly patients with large tumors, but also includes younger patients with smaller tumors, located at areas where cosmetic outcome is an important factor, such as the nose. Irregularities or disfigurements after skin cancer treatment may, therefore, lead to social and psychologic problems [11, 12].

Aside from being more prone to social and psychologic problems, complex skin cancer patients are usually treated by a multidisciplinary team of dermatologists, plastic surgeons, and radiation oncologists as part of a step-by-step process. The patient (preferences) and the lesion must first be assessed to assure that surgery is the best treatment [13]. If surgery is the preferred option, the specific method is chosen depending on the type and size of the lesion [13]. Usually, dermatologists remove the tumor with Mohs micrographic surgery (MMS) in case of BCC or cSSC or by means of a staged micrographic surgery technique (Breuninger) in case of LM [13–17]. Subsequent reconstructions may be more challenging, requiring the expertise of a plastic surgeon [13]. In addition, some patients require adjuvant radiotherapy. This step-by-step process, involving multiple healthcare providers, may complicate the care process for this patient group.

High-quality care should ideally be tailored to the needs of individual patients (i.e., patient-centered care) [18, 19]. A qualitative systematic review focusing on the needs and experiences of skin cancer patients revealed the scarcity of qualitative studies regarding this subject [20]. Existing literature on patients with complex skin cancer has predominantly focused on surgical techniques [21, 22]. Knowledge about complex skin cancer patients’ experiences and needs regarding their care is currently lacking.

The aim of the current study was to explore the experiences and needs of patients who had undergone surgical treatment by a dermatologist for BCC, cSCC or LM with a subsequent reconstruction by a plastic surgeon. The results of this study can be used as input to facilitate patient-centered care for complex skin cancer patients by tailoring care to their needs.

## Patients and methods

### Study design

A qualitative interview study among complex skin cancer patients was conducted. Qualitative research is most suitable for gaining an in-depth understanding of patients’ experiences and needs [23, 24]. Individual interviews rather than focus groups were used because the average age of complex skin cancer patients is high and some of them were affected by disabling hearing impairment. In addition, for some patients, skin cancer is a sensitive subject, which might prevent them from speaking freely about their disease in focus groups [25].

### Study setting

The study took place at Erasmus MC in Rotterdam, the Netherlands. This is an academic tertiary referral center for skin cancer patients and among the largest MMS centers in Europe with approximately 1700 MMS procedures annually. Approximately, 10% of patients treated with MMS require reconstruction by a plastic surgeon, usually under general anesthesia. Therefore, a special outpatient clinic is present at this center for patients who need to be evaluated by both a dermatologist and plastic surgeon.

### Study sample

Electronic patient files were screened to select patients older than 18 years who had been to the special outpatient clinic mentioned above. We consecutively included patients who underwent surgical treatment within the preceding year by a dermatologist, followed by a reconstruction by a plastic surgeon for a BCC, cSCC or LM. Patients were excluded if they had other types of skin malignancies or if they were not able to speak Dutch. Data regarding gender, age, skin cancer type and location, type of treatment and method of reconstruction were collected from the electronic patient files.

Eligible patients were sent a letter containing study information and an invitation to participate in a 30-min interview directly before or after their already planned follow-up consultation. If there was no more consultation planned, patients were asked to be interviewed by phone. After 2 weeks, a reminder was sent. If another person was present during the consultation (e.g., caretaker or partner), this person was also invited to join the interview to include their perspective.

We used purposive sampling, i.e., we explicitly selected information-rich cases to answer our research question by including a variable sample of patients in terms of sex, age and diagnosis [26]. Participant recruitment ended after data saturation was reached, which was the case when there were no new code (groups) created.

### Data collection

Sixteen interviews were held; 13 were conducted face-to-face and three by telephone. A topic guide, based on previous research of the authors, expert opinion and information derived from the literature, was used to structure the interviews (see Appendix 1) [27–30].

The first three interviews were conducted by two researchers (M.H. and S.v.E), the remaining interviews were held by one (M.H.). The interviewers were not involved in the medical care of the interviewed patients. Interviews started with the explanation that everything would be analyzed anonymously and stimulated free expression of opinions. All sessions were audio-taped, transcribed verbatim and anonymized.

### Data analysis

An inductive approach to data analysis was used allowing meaning to emerge from the data, rather than from pre-determined categories [31]. Two researchers (S.v.E. and M.H.) independently openly coded the first four transcripts, using the qualitative data analysis software ATLAS.ti (Version 8) [32]. These codes were discussed with a third researcher (M.L.) and adjusted if necessary, which resulted in a preliminary coding scheme. Next, all transcripts were coded using this coding scheme by one researcher (M.H. or S.v.E), then checked by the other.

Interpretive and iterative constant comparison followed the initial coding phase, in which different codes were compared and the relationship between codes was explored to detect emerging themes. The overall analytical process resulted in the identification of main themes and sub-themes regarding the experiences and needs of patients with complex skin cancer.

### Ethical considerations

The medical ethics committee of Erasmus MC declared that the Medical Research Involving Human Subjects Act did not apply to the present study (MEC-2018-1677). All participants provided written informed consent and participation was voluntarily. This study has been designed and is reported in accordance with the SRQR (Standards for Reporting Qualitative Research) recommendations [33].

## Results

### Patient characteristics

The median age of the participants (7 women and 9 men) was 71.5 years (range 47–87). Six patients were diagnosed with BCC, five with cSCC and five with LM. Further tumor and treatment details are described in Table 1.

**Table 1:** Characteristics of participating patients

Patient	Age	Sex	Diagnosis	Location	Treatment	Method of reconstruction	Interview
1	87	Male	LM	Cheek	Staged excision	FTSG from supraclavicular	Individual
2	71	Male	cSCC	Forehead	Re-excision and ART	Free skin grafted muscle flap	With partner
3	87	Male	cSCC	Scalp	Re-excision and ART	Free skin grafted muscle flap	With partner
4	65	Female	BCC	Nose	MMS	FTSG from preauricular	Individual
5	78	Male	cSCC	Scalp	MMS and ART	Free skin grafted muscle flap	Individual
6	73	Female	cSCC	Lower leg	Staged excision	SSG from upper leg	Individual
7	47	Female	cSCC	Lower leg	Staged excision	SSG from upper leg	Individual
8	67	Male	LM	Fifth digit of hand	Staged excision	FTSG from groin	With partner
9	51	Female	LM	Nose	Staged excision	Local bilobed flap	Individual
10	80	Male	LM	Vertex	Staged excision	Secondary intention*	Individual
11	52	Male	BCC	Nose	MMS	FTSG from preauricular	Individual
12	72	Male	BCC	Nose	MMS	Local hatchet flap	With partner
13	64	Female	BCC	Nose	MMS	Paramedian Forehead Flap	Individual
14	78	Female	BCC	Nose	MMS	FTSG from groin	Individual
15	55	Female	LM	Nose	Staged excision	FTSG from preauricular	Individual
16	72	Male	BCC	Cheek	MMS	Local advancement flap	With partner

*ART* adjuvant radiotherapy, *MMS* Mohs micrographic surgery, *LM* lentigo maligna, *cSCC* cutaneous squamous cell carcinoma, *BCC* basal cell carcinoma, *FTSG* full-thickness skin graft, *SSG* split-thickness skin graft

^*^Was initially planned for SSG and tissue expander, but reconstruction was postponed

### Complex skin cancer patients’ experiences and needs

**Fig. 1:**
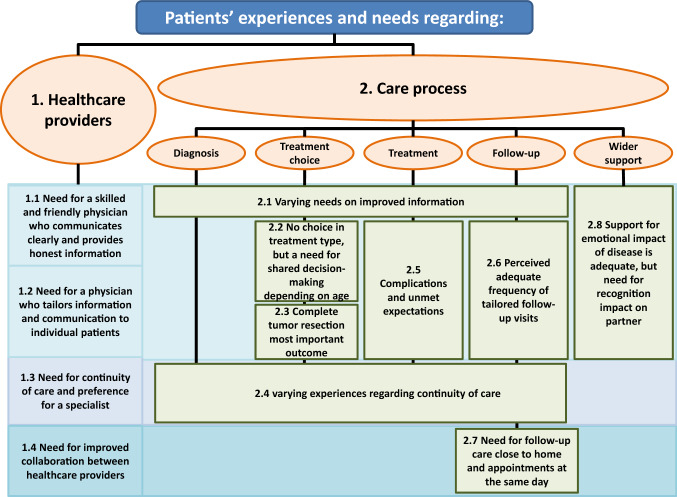
Overview of identified themes and sub-themes on the experiences and needs of complex skin cancer patient

Based on the patient interviews, twelve sub-themes were identified on the experiences and needs of complex skin cancer patients (Fig. 1).

### Patients’ experiences and needs regarding healthcare providers

#### Need for a skilled and friendly physician who communicates clearly and provides honest information

Patients emphasized the importance of a physician who communicates clearly and provides honest information throughout the entire process of care (Table 2). In this way, they fully know where they stand and what to expect. They need to trust their physician and the provided information. In addition, they reported the importance of physicians to be skilled, but also to show compassion and to be friendly.

**Table 2:** Illustrative quotes on need for a skilled and friendly physician who provides clear and honest information

*“And be honest, don’t make it worse, don’t make it less serious. Just say it as it is.”* **Patient 11 (52-year-old male)**	
*“The kindness of the people, of the physicians [is most important]. And their expertise”* **Patient 14 (78-year-old female)**	

#### Need for a physician who tailors information and communication to individual patients

Patients expressed the importance of information and communication to be tailored to individual patients’ needs (Table 3). They suggested that physicians should ask each patient whether he/she needs more information and adjust the information provision accordingly. Furthermore, patients indicated that physicians should adjust their explanation to the particular patient to make sure every patient understands.

**Table 3:** Illustrative quotes on need for a physician who tailors information and communication to individual patients

*“But I wasn’t asked whether I needed more information.* (…) *It’s never a bad thing to hear that”* **Patient 15 (55-year-old female)**	
*“That they explain things in a clear, understandable way to the patient. Sometimes they are talking to clinicians and other occasions, with all due respect, they are talking to pavers. They must explain things clearly to both of them and that is a matter of choosing the right words.”* **Patient 11 (52-year-old male)**	

#### Need for continuity of care and preference for a specialist

Patients generally expressed the need to be seen by the same physician during diagnosis, treatment and follow-up visits (Table 4). This ensures them that their physician has all relevant information and also strengthens the bond with their physician. In addition, patients reported to prefer to be treated by a specialist instead of a physician assistant (PA), because they feel he/she is the expert. Patients particularly wished to be treated by a skilled expert, as their skin cancer is often located in the face and they wanted it to be done neatly. Nevertheless, patients generally stated it to be acceptable if PAs would provide information and explain things about the treatment process.

**Table 4:** Illustrative quotes on need for continuity of care and preference for a specialist

*“You know what also disappointed me, it suddenly comes to mind, I think we drove to Rotterdam about seven times and every time I was seen by another person. Instead of one physician who would treat me. At a certain point you have a bond with someone and then suddenly you are seen by someone else.”* **Patient 8 (67-year-old male)**	
*“I really wanted the specialist to do that. (…) That it really had to be done by the specialist himself because after all it is my face.”* **Patient 15 (55-year-old female)**	

#### Need for improved collaboration between healthcare providers

Patients expressed the need for improved collaboration between healthcare providers and between hospitals (Table 5). They noticed that healthcare providers sometimes communicate past each other and are not aware of important information. They indicated to sometimes receive wrong information due to miscommunication, such as a wrong dismissal date. A national electronic patient file for all hospitals was suggested to improve communication.

**Table 5:** Illustrative quotes on need for improved collaboration between healthcare providers

*“That things get mixed up now and then (…) Yes, this is not necessarily just about me… I notice this in general. They [dermatologist and plastic surgeon] also say that about each other. That things do not go smoothly.”* **Patient 7 (47-year-old female)**	
*“Well there was some confusion because the plastic surgeon said that I could go home after I had been treated, but the nurse on the ward said that I had to stay overnight.”* **Partner of patient 8 (Partner of 67-year-old male)**	

### Patients’ experiences and needs regarding the care process

#### Varying needs on improved information

Whereas many patients indicated to be satisfied with the information they received, a need for improved information was also often reported (Table 6). This applied to all phases of the care process. Some patients indicated that they received hand-outs in addition to oral information and some patients were also shown pictures of other complex skin cancer patients. These pictures were regarded as informative by some patients; whereas, others preferred not to see them and reported that physicians should at least warn patients beforehand. Patients often searched the internet for additional information themselves, but as this was sometimes experienced as shocking, they generally preferred to receive clear information on hand-outs from physicians. Overall, patients emphasized the importance of written information besides oral information, because they were not able to remember all information provided during consultation. This was specifically the case for follow-up care.

**Table 6:** Illustrative quotes on varying needs on improved information provision

*“Yes, that they ask if you want to see pictures and that they can be shocking. That they would warn you in advance. But I had already seen them and that was not a disaster in itself but if I were given the choice I would rather not have seen them”* **Patient 4 (65-year-old female)**	
*“I still Googled a bit at home but not too much because it doesn’t make you feel well.”* **Patient 4 (65-year-old female)**	
*“Yes, you leave and that’s it. Yes, the medical world knows more than a layman. Then you could put a resume on a piece of paper and pass it on. So you have something tangible.”* **Patient 1 (87-year-old male)**	
*“You know, I have had so many things, at a certain point I just let it happen.”* **Patient 6 (73-year-old female)**	

#### No choice in treatment type, but a need for shared decision-making, depending on age

Overall, patients mentioned that they were not given a choice in treatment (Table 7). They did not consider this as a problem, because they fully trusted the physician in choosing the best treatment. Some patients stated that they were told what would happen if their tumor would not be treated, but receiving no treatment was never a real option for patients: they came all the way from a general hospital to have their skin cancer removed. Some patients appreciated that they were able to decide on type of reconstruction and between local or general anesthesia.

**Table 7:** Illustrative quotes on no choice in treatment type, but a need for shared decision-making, depending on age

“*With me it was actually the case that there was never any choice. It had to be removed and that was it”* **Patient 7 (47-year-old female)**	
*“That physicians mention a few options. I can imagine that old people don’t really like this, but I do like to hear them. So they can include me in their thoughts and decision-making. There might also be people who will just go along with things, but I am not like that.”* **Patient 11 (52-year-old male)**	
*“Yes, they are specialists, I am no expert so I don’t know. They told me that this would be the best solution. I just trust them, because of their experience.”* **Patient 10 (80-year-old male)**	

Specifically younger patients expressed the need to be involved in the decision-making process and preferred to discuss treatment choices if available. They stressed the importance of being informed about all treatment options including the benefits and disadvantages. As such, they are able to make an informed decision together with their physician. On the contrary, older patients generally stated to fully trust their physician in making the decision, as he or she is the expert.

#### Complete tumor resection most important outcome

Patients indicated that they considered the complete removal of the tumor to be more important than the cosmetic outcome (Table 8). They reported to be scared of recurrence and, therefore, found it most important that it was completely removed, regardless of the scar size. Still, they preferred the scars to be as small as possible. They preferred surgery opposed to radiotherapy, because surgery confirms complete clearance of the tumor. Furthermore, patients expressed a preference for the skin cancer to be removed as quickly as possible to prevent it from growing further. Improvement of quality of life was also mentioned as an important outcome.

**Table 8:** Illustrative quotes on complete tumor resection most important outcome

*“Even though it won’t be perfectly beautiful, and it will never be. There is not much more to improve. I don’t really mind that spot and that scar, as long as I look a little presentable.”* **Patient 12 (72-year-old male)**	
*“Although the cancer might not be completely gone (…) my quality of life has indeed improved and that is important to me.”* **Patient 13 (64-year-old female)**	

#### Varying experiences regarding continuity of care and type of healthcare provider

Patients reported various experiences regarding continuity of care during the entire care process (Table 9). Some patients were seen by the same healthcare provider every time, whereas others reported to have seen a different physician on each occasion. Seeing multiple physicians made them feel that the physicians were not really involved in their care, even if they prepared the consultation well. Overall, patients reported to be satisfied with the received care by medical specialists. In general, patients did not like to be treated by PAs or residents instead of specialists, particularly if they had not given permission for this.

**Table 9:** Illustrative quotes on varying experiences regarding continuity of care and type of healthcare provider

*“I had two people at my bedside who were both physicians, but who didn’t have a clue what kind of patient they had in front of them*” **Patient 2 (71-year-old male)**	
*“Then you get a different one every week. Even a PA once, I had not given permission for her to do the procedure on me. And then I immediately said that I did not want that. (…) At one point, I had the same surgery assistants three times in a row. That's really great.”* **Patient 15 (55-year-old female)**	

#### Complications and unmet expectations

Some patients mentioned that they had experienced complications such as bleeding, infections and pain (Table 10). They stated that their treatment went better than expected, but the time until full recovery was disappointing. After having been shown pictures of the expected result and receiving explanation of the expected scar size, patients still reported that their scar turned out larger than expected. It also bothered patients that their scar sometimes frightened other people. According to patients, improved information and explanation beforehand could facilitate being properly prepared for potential complications.

**Table 10:** Illustrative quotes on complications and unmet expectations

*“Yes, at first I thought it wouldn’t be too bad, but it takes a long time and I hope that this will only get better. Applying drops and ointment every day, that is quite challenging”* **Patient 15 (55-year-old female)**	
Partner of patient: “*Yes, it shocks people.”* Patient: *“Recently we were visiting some people and the first thing they said is what is that and what have you done? That is of course not very pleasant.”* **Patient 3 (87-year-old male)**	

#### Perceived adequate frequency of tailored follow-up visits

Patients generally reported to be satisfied with the frequency of the follow-up checks by their dermatologist and plastic surgeon (Table 11). Some patients expressed the need for an increase or decrease of the interval time between visits. Most patients, however, stated they could adjust the frequency according to their needs. Patients experienced the hospital to be easily accessible; if they noticed new lesions in between follow-up visits, they could come by right away. During follow-up visits, they preferred a physician to perform a full body skin examination as they lack the expertise to self-examine their skin adequately.

**Table 11:** Illustrative quotes on perceived adequate frequency of tailored follow-up visits

*“If I want to come more often, I’m able to do so (…) when I call I can come by immediately”* **Patient 7 (47-year-old female)**	
*“She said, and I agree with her, ‘I expect an active attitude from the patient, when you see spots yourself, you keep an eye on them’.”* **Patient 11 (52-year-old male)**	
*“Obviously, as a layman you can’t see whether there are any more bad spots that have not been removed.”* **Patient 1 (87-year-old male)**	

#### Need for follow-up care close to home and appointments at the same day

Patients who live far away from the hospital stated that they preferred to have follow-up visits in a hospital closer to their home to minimize their traveling time (Table 12). Specifically, elderly patients reported the need for hospital visits to be scheduled at the same day to decrease the number of hospital visits. They also mentioned to be bothered with the high parking costs which were accompanied by the follow-up visits.

**Table 12:** Illustrative quotes on need for follow-up care close to home and appointments at the same day

*“I was actually referred back to [name hospital], but that was also my own choice because I thought they could check me there just as well.”* **Patient 9 (51-year-old female)**	
*“I don’t know, when she says you have to come then or then, I just come again. But yesterday we also visited two [specialists] and now I am here again. So preferably as many consecutive visits as possible.” (…) “Yesterday 6.5 euros [parking costs], last week again 6.5 euros. We are only old age pensioners.”* **Patient 2 (71-year-old male)**	

#### Support for emotional impact of disease is adequate, but need for recognition impact on partner

Patients indicated the whole process to be intense (Table 13). Some patients reported they became more emotional and more ashamed because of the impact of the treatment and disappointing recovery and scars. Despite the emotional impact of the disease, patients indicated not to require psychological care, although this was offered to them. Instead, they preferred to talk to friends or their primary care physician about it.

**Table 13:** Illustrative quotes on support for emotional impact of disease is adequate, but need for recognition impact on partner

*“I became much more emotional after the operation. I've never had that before. My kids also said, ‘I don’t really recognize my father like that’.”* **Patient 2 (71-year-old male)**	
*“No. I have my own network, both friends and colleagues and privately. So no, I didn’t feel the need to talk to someone else about it.”* **Patient 11 (52-year-old male)**	
*“It’s really difficult for the partners. People often forget about this. If you want another area for improvement: more attention for the partner”* **patient 5 (78-year-old male)**	

Patients also expressed the need for recognition and attention of the impact of the disease on partners, as it may be difficult for them to cope with. For example, it might be easier for patients to accept the risks of high-risk surgery than for their partner. Providing more attention to partners of patients in the entire care process was, therefore, suggested.

## Discussion

This study focused on the experiences and needs of complex skin cancer patients, who had undergone surgical treatment by a dermatologist and subsequent reconstruction by a plastic surgeon, and revealed a range of themes which could be used as input to organize patient-centered care for this unique patient group.

Several needs regarding healthcare providers emerged which are reflected in patients’ experiences and needs regarding the entire care process. Consistent with various previous studies, both in- and outside the field of (skin)cancer, patients emphasized the importance of a friendly physician who provides clear and honest information [34–39]. Particularly, the need for clear information seems a profound need among patients. Although patients in our study were generally satisfied with the provided information, the need for improved information (provision) reflects through all phases of the care process. They suggested to provide more comprehensive written information, which is currently being implemented in our department. Aside from receiving clear and honest information, the need for physicians to tailor information and communication to individual patients was identified. To enhance patient-centered care, it is, therefore, advocated to improve information provision and to adapt it to individual patients. This could be achieved by improving communication skills (e.g., increased focus on shared decision-making) in the medical curriculum and using tools such as question prompt lists or patient-reported outcome measures [40–42].

Complex skin cancer patients also expressed the need for continuity of care and improved collaboration between healthcare providers. A need for continuity of care, defined as a continuous caring relationship with a healthcare provider, was also identified in qualitative studies focusing on (non-complex) skin cancer patients [27, 43]. According to patients, this strengthens the bond with their physician. Continuity of care is associated with various positive outcomes including decreased chance of hospitalization, costs reduction and improved compliance with medical regimes [44–47]. The need for optimal collaboration between healthcare providers may not be surprising as their care process is a complex step-by-step process, involving multiple healthcare providers. According to patients, this process could be improved, as they experienced that healthcare providers sometimes communicate past each other. Related to this, patients indicated to prefer multiple follow-up visits of different medical specialties to be planned on the same day, which also demands effective collaboration between medical departments.

With respect to experiences and needs regarding the care process, complex skin cancer patients had different needs regarding shared decision-making. Especially younger patients preferred to be involved in treatment decisions. On the contrary, elderly patients preferred the physician to make the decision for them, as they believed the physician is the expert. This is consistent with previous studies indicating older patients are less likely to participate in shared decision-making [48, 49]. Being able to adjust the frequency of follow-up visits to a patient’s own needs was also experienced positively by patients and probably contributes to their satisfaction with the frequency of follow-up visits. Furthermore, patients recognized the high emotional impact of the disease. Whereas they considered their own received support as adequate, they emphasized the need for recognition of the impact of the disease for their partner. It is, therefore, advocated for physicians to be sensitive to the needs for psychological support of both patients and their partners, as partners are known to be an essential source of social support for patients [50]. Improved collaboration between healthcare providers of different disciplines, such as medical specialists, social workers and psychologists, has demonstrated to facilitate the identification of unmet physical and psychosocial needs [11, 12, 51].

Although cosmetic outcome is also important, the most important aspect for complex skin cancer patients is that the tumor is completely removed. However, patients also reported complications and unmet expectations. Even after seeing pictures before the surgery, patients did not expect the size of the facial scars to be that large. Previous research already has revealed a gap in the communication between surgeons and patients about the expectations of scarring due to surgery [52]. Discrepancies in expectations could be addressed in guidelines to educate surgeons on the impact of (even minor) facial scars to patients [52]. It also, once more, emphasizes the importance of improved information and communication.

Results of this study imply that to improve patient-centered care for complex skin cancer patients, information, communication, as well as wider care aspects should be tailored to individual patients and their partner. This is in line with recent trends of individualizing care based on the individual needs of patients. Besides improving information and communication skills of physicians, shared decision-making tools (decision aids) can be used in the care process [53, 54]. In addition, healthcare applications (apps) may be used to stimulate personalized information provision for patients. As far as these apps are integrated within the care pathways, they may also facilitate personalized (follow-up) care and improve coordination between healthcare providers [55, 56]. Several studies have shown that patient-centered or personalized care improves patient experiences and outcomes [57, 58].

A limitation of this study was that we only interviewed patients of one academic hospital. Although qualitative research is always context specific [59], the generalizability of our findings increases as we reached maximum variation in our sample of patients in terms of relevant characteristics (e.g., age, sex and diagnosis). A strength of our study is that our qualitative study, using a thorough methodology, to our knowledge is the first study providing an in-depth understanding of the experiences and needs of patients with complex skin cancer.

In conclusion, the current study provides insight into the experiences and needs of complex skin cancer patients and provides suggestions to improve patient-centered care. Continuity of care and improved collaboration between heath care providers is essential for this group of patients. Furthermore, given the differences in experiences and needs within these patients, it is advocated for physicians to be sensitive to the individual needs of patients and adapt their information, communication and care accordingly. This should not be limited to the walls of the hospital, but also include the wider context, for instance by also focusing on interdisciplinary collaborations and by offering psychological support to partners of patients.

## Data Availability

Raw audio and transcribed material (in Dutch) are available upon request.

## Introduction

- Introduction with background, aim of the study and structure of the interview.
- Checking informed consent form, permission for audio-taping.

## General

- General experiences with received care.

## Diagnosis

- Experiences and needs regarding diagnostic process.
- Impact of diagnosis.
- Experiences and needs regarding healthcare providers during diagnostic process.
- Experiences and needs regarding information during diagnostic process.
- Suggestion for improvement regarding the diagnostic process.

## Treatment (choice)

- Experiences and needs regarding (choice of) treatment.
- Treatment expectations.
- Experiences and needs regarding treatment providers.
- Experiences and needs regarding information during treatment.
- Suggestions for improvement regarding treatment (choice).

## Follow-up

- Experiences and needs regarding follow-up.
- Experiences and needs regarding providers during follow-up care.
- Experiences and needs regarding information during follow-up.
- Suggestions for improvement regarding follow-up.

## Closing

- Patient initiated additional topics.
- Thanking and closing.
